# Positional Differences in Decision-Making Situations during Professional Rugby League Match-Play

**DOI:** 10.5114/jhk/186559

**Published:** 2024-05-17

**Authors:** Lily Turek, Kenji Doma, Wade Sinclair, Jonathan Connor

**Affiliations:** 1 Sport and Exercise Science, College of Healthcare Sciences, James Cook University, Townsville, Australia.; 2 Orthopeadic Institute of Queensland, Townsville, Australia.; 3 North Queensland Cowboys Rugby League Football Club, Australia.

**Keywords:** group dynamics, performance analysis, affordances, constraints, coaching

## Abstract

The aim of this study was to explore the types and frequency of decision-making situations of rugby league players during defensive situations and examine whether they were predictive of key performance indicators (KPI). Fifteen elite rugby league matches were coded using notational analysis methods. Specific defensive situations were analysed, including the number of: one-on-one situations with an opposing attacker (1-on-1), two-on-one situations (2-on-1), and combined 1-on-1 and 2-on-1 situations (i.e., total decisions; TDs). There was no relationship between the game outcome and game KPIs for TDs or 1-on-1 decision-making situations. However, successful tackles and missed tackles were predictive of 2-on-1 decision-making situations. Positional differences revealed that back rowers were exposed to the greatest number of decision-making situations, while wingers had the lowest exposure. The total number of decisions and the number of 1-on-1 decisions made by the centres and wingers were significant predictors of line breaks. Additionally, 2-on-1 decisions were significant predictors of line breaks for backrowers. The findings of this study suggest that the type and frequency of decision-making situations in Rugby League are position specific. Practical applications for coaches are discussed to ensure that practice approaches are representative of the various defensive decision-making demands players experience during a game, based on their position.

## Introduction

Performance analyses of invasion game-type sports are commonly underpinned by the examination of key performance indicators (KPIs) to describe the outcome of a game. Depending on the demands of the game, KPIs can include a combination of activity profiles, technical, as well as tactical abilities/outcomes (Austin and Kelly, 2013; Cummins and Orr, 2015; Gabbett, 2005; Sampaio and Janeira, 2003). Within rugby league, performance indicators such as tackling, scrummages and high-speed contacts with opposing players have all been associated with successful game performance (Hughes and Bartlett, 2002; Hulin and Gabbett, 2015; Johnston et al., 2014; Kempton et al., 2014). However, a notable inadequacy of KPIs is the inability to take into account contextual information about the game performance.

For example, performance analysis of KPIs often involves recording missed tackles and technical errors (e.g., dropped balls, forward passes etc.); albeit the underlying cause of why these errors occurred remains unclear (Gabbett, 2013; Hulin and Gabbett, 2015). Understanding the mechanistic causes of these errors is further complicated within invasion-based team sports, given the dynamic nature of players’ roles differing between positions, and during attacking and defensive phases of the game. Sirotic et al. (2011) reported that players’ positions such as forwards (categorised as prop and second row) run the ball on more occasions during offensive plays, and complete more tackles per minute during defensive plays, when compared to backs (categorised as wingers and centres). Given the varying physical and tactical demands between positions and attacking or defensive phases of the game, it is unsurprising that KPIs would also differ among these contextual factors. Kempton and Coutts (2016) highlighted that defensive performance was generally associated with increased physical demands during rugby league gameplay, while achieving good defensive technical KPIs, such as tackling to stop the opposition scoring or gaining additional meters, were linked with greater game success (Hulin et al., 2015). Although positional roles, offensive and defensive behaviours all play a role in KPIs, there has been limited investigation into the underpinning factors that contribute to successful or unsuccessful KPIs, particularly within defensive performances.

A recent and increasingly prominent framework to understanding team sport expertise is grounded in the dynamic interaction between the specific individual and environmental constraints (Davids et al., 2013). According to this approach, defensive players in Rugby League can be viewed as acting collectively to: 1) identify and mark (i.e., to couple defensive behaviour with) an opposing attacker; 2) commit to (and successfully perform) a tackle; and 3) avoid a line break occurring (Gabbett and Abernethy, 2013). Creating situations whereby the defensive player must identify and mark two offensive players, also referred to as a ‘draw and pass’ situation, is a common offensive tactic to increase the likelihood of a defensive error such as an unsuccessful tackle or line break. Gabbett and Abernethy (2013) previously investigated a type of 2-on-1 defensive decision-making (DM) situations in elite rugby league players during a temporal occlusion task, noting significant attentional demands placed on defensive players using a dual task paradigm. As the defender must choose between tackling two offensive players with one carrying the ball, these 2-on-1 defensive situations are arguably more complex than 1-on-1 defensive situations. Furthermore, during a 2-on-1 defensive situation, the opposing attacker would likely have a greater number of possible affordances (i.e., possible actions in which to execute) with their defensively unmarked teammate. While this defensive situation has previously been examined within a laboratory setting, no study to date has examined whether these DM situations are associated with defensive KPIs during actual rugby league gameplay.

There is currently scarce research investigating how complex game situations impact upon the performance outcome. Understanding the impact of opposing teams attempting to outnumber the defensive team is one such a game situation considered critically important anecdotally, yet scarcely researched empirically. It is also important to consider the positional specificity of these DM situations with regard to defensive KPIs. Therefore, the aim of this study was to examine the impact of positional DM during defensive plays on game performance in rugby league. It was hypothesized that the number of DM situations key defenders were exposed to would have a direct impact on match performance factors. Furthermore, it was hypothesized that the greater number of DM situations would predict unsuccessful defensive technical performance.

## Methods

### Matches

From the 15 selected matches, a total of 492 sets and 2,741 match-related events, generated specifically by edge defenders, were coded. Edge defenders were chosen for the purpose of this analysis given their defensive roles during a rugby league game, in which they are more susceptible to an attacking opposition attempting a number of deceptive manoeuvres to try and evade or deceive the defensive line. All analyses were conducted during defensive situations with a focus on the type of DM situations and the KPI’s of edge defenders during game play. The positions of edge defenders included the wing (positioned closest to the sideline), the centre (positioned between the wing and the halfback), the halfback (positioned between the centre and the backrower) and the backrower (positioned closest to the centre of the field, of all four positions).

### Design and Procedures

This study was conducted across three stages. Firstly, National Rugby League (NRL) games over the 2017 competitive season were put through a random number generator from Round 1 to 30 to select 15 NRL games for subsequent analysis. Secondly, a decision-making criterion was developed with the assistance of elite rugby league coaches (n = 3) and sport scientists (n = 3), to determine the relationship between decision-making and performance outcomes. Finally, the decision-making criterion was applied and assessed during 15 randomly selected 2017 NRL games. Analysis software (Analyzer, The League Analyst, Version V4, Fair Play Pty Ltd, Brisbane, Australia) was used to conduct hand notational analysis by coding video footage of each DM situation in matches. The analysis software optimised viewing of video footage by allowing the analyst to pause, rewind and watch each play in slow motion. All video footage was obtained and viewed through the analysis software. The study obtained clearance by the Institutional Human Research Ethics Committee (James Cook University, protocol code: H7515; approval date: 14 September 2018) and was conducted in line with the Declaration of Helsinki.

### Measures

#### Identification, Selection and Definition of Variables

All variables analysed in this study represented important aspects of DM situations and match-specific outcomes. Through consultation with elite NRL coaches and sport scientists, the variables were separated into three categories, including: 1) complexity of DM; 2) match-performance factors; and 3) defensive phases. The complexity of DM situations was classified as the total number of DM situations (TDs) which combined 1-on-1 and 2-on-1 DM situations. The 1-on-1 and 2-on-1 DM situations required the edge defender to either respond to one opposing player (i.e., 1-on-1) or two opposing players (i.e., 2-on-1) in the passage of play immediately before them on the playing field. This classification was based on the notion that an increased number of opposing players would exacerbate the complexity of DM situations for defenders due to greater opportunities for a defender’s action (Passos et al., 2008). Match-performance factors consisted of points conceded (i.e., the number of points allowed by the opposing team), successful tackles (i.e., the number of successful tackles made by the defending team), missed tackles (i.e., the number of missed tackles for every attempted tackle made by the defending team), attempted tackles (i.e., the number of tackles attempted by the defending team) and offensive handling errors (i.e., the number of errors made by the opposing team).

The defensive phases, which were manually coded, included line breaks (i.e., the number of times the opposing team successfully broke the defensive line), committed actions (i.e., the number of times defensive players committed to executing a defensive action against an opposing player) and match-up (i.e., the number of times defensive players were approached by opposing players executing attacking manoeuvres). These variables were also analysed per position (wing, centre, halfback and backrower) and averaged between the left and right sides. For example, the number of line breaks that occurred during a match because of a winger’s DM situation was averaged between the left and right wingers.

#### Quantification of Decision-Making

Similarly to the definition of the variables described in the earlier section, the method of determining DM situations was designed by the same group of coaches and sport scientists. Accordingly, four categories were developed to determine whether DM opportunities occurred during game play. A defensive DM opportunity was established when defenders appeared to make an attempt to read an attacking play, followed by an appropriate response to act upon (thus, making a decision). For example, if two offensive players appeared to accelerate towards a defensive player, with one offensive player carrying the ball, then the defensive player would be expected to make a decision to either defend the ball-carrier or to tackle another offensive player from receiving the ball. According to the complexity of DM situations, 1-on-1 was classified when one defensive player faced one offensive player and 2-on-1 was classified when one defender had to choose between tackling the offensive player carrying the ball or the support offensive player that could receive the ball by a short pass. The categories to address these DM opportunities were based on previous game data to rationalize the definitions of each DM category. All DM performance opportunities were well suited to at least one DM situation, allowing it to be a valid and reliable criterion.

### Statistical Analysis

All data were analysed using the Statistical Package of Social Sciences (SPSS, version 24, IBM Corp., Armonk, N.Y., USA) with an alpha level set at ≤ 0.05 and descriptive information expressed as mean ± standard deviation. For comparisons in the number of decisions made for each type of the decision (i.e., TDs, 1-on-1 and 2-on-1) among positions, a univariate analysis of variance was conducted with each decision as a dependent variable and the positions as random factors. Post-hoc analysis was conducted using Bonferonni’s pairwise comparison to determine the location of the difference. Effect sizes (ES; Cohen’s *d*) with 95% confidence intervals (CIs) were also calculated to examine the magnitude of differences in the number of decisions made between positions for each type of the decision, with 0.2 considered as a small ES, ≥0.5 as a moderate ES, and ≥0.8 as a large ES (Cohen, 1988). To determine whether match performance indicators (i.e., successful tackles, missed tackles and offensive handling errors) predicted the number of decisions made for each type of the decision (i.e., TDs, 1-on-1 and 2-on-1), the data for each match were separated for each position, which generated 60 data sets, based on 15 matches across four separate positions (wing, centre, halfback and backrower). Subsequently, generalized estimating equations (GEE) were applied, with the positions as a repeated measures variable, the type of the decision as a dependent variable and match performance indicators as predictor variables. A generalized linear mixed model was used to ascertain whether the positions (i.e., wingers, centres, halfbacks and backrowers) predicted the match-phases (i.e., line breaks, committed actions and match-ups), with the matches treated as a random effect variable and match-phases as the binary outcome. To assess for assumptions, the variance inflation factor (VIF) measures of the regression model was below 5 (Akinwande et al., 2015) for the predictor variables, and the critical values of the Durbin Watson statistic were between 1.5 < d < 2.5 (Uyak et al., 2007), suggesting that the predictors exhibited acceptable multi-collinearity.

## Results

**Figure 1:**
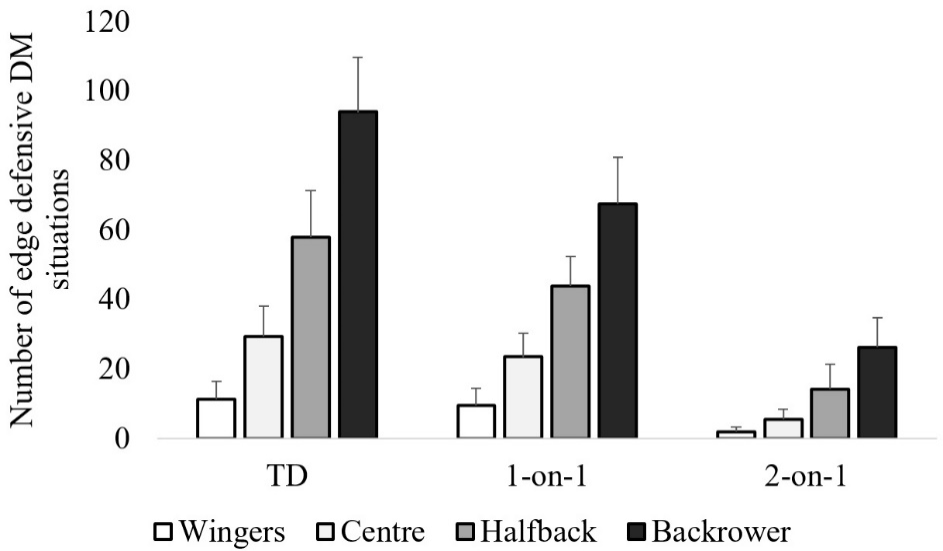
The mean ± standard deviation of the number of total decisions made (TDs), 1-on-1 situations and 2-on-1 situations with comparison between positions cumulatively across games. * No significant differences (p > 0.05), with all other comparisons being significantly different (p < 0.01)

Figure 1 depicts differences in the number of decisions made among positions. Accordingly, back rowers exhibited a significantly greater number of decisions (i.e., TDs, 1-on-1 and 2-on-1) than wingers (*p* < 0.01), centres (*p* < 0.01) and halfbacks (*p* < 0.01) with a large effect size (1.54–7.07; Table 1). Similarly, halfbacks made a significantly greater number of decisions than centres (*p* < 0.01) and wingers (*p* < 0.01) with a large effect size (1.60–4.97; Table 1). Finally, centres made a significantly greater number of TDs and 1-on-1 DM situations than wingers (*p* < 0.01) with a large effect size (2.34–2.57; Table 1). However, no significant differences were found among these positions for 2-on-1 DM situations (*p* > 0.05), although the effect size was large (1.57; Table 1).

**Table 1:** Effect size calculations with associated 95% confidence intervals (CI) between each position for the number of total decisions made (TDs), 1-on-1 situations and 2-on-1 situations

Position Comparison		TD (95% CI)	1-on-1 (95% CI)	2-on-1 (95% CI)
Wingers vs.	Centre	2.57 (1.55–3.46)	2.34 (1.35–3.19)	1.57 (0.71–2.34)
	Halfback	4.58 (3.12–5.79)	4.97 (3.42–6.26)	2.39 (1.40–3.25)
	Backrower	7.07 (5.00–8.77)	5.80 (4.05–7.24)	4.02 (2.69–5.14)
Centre vs.	Halfback	2.52 (1.51–3.40)	2.64 (1.60–3.54)	1.60 (0.74–2.37)
	Backrower	5.10 (3.51–6.40)	4.16 (2.80–5.30)	3.28 (2.11–5.27)
Halfback vs.	Backrower	2.46 (1.46–3.33)	2.11 (1.17–2.94)	1.54 (0.69–2.31)

With respect to whether match-performance measures (i.e., successful tackles, missed tackles and offensive handling errors) predicted each decision made (i.e., TDs, 1-on-1 and 2-on-1), successful tackles predicted TDs (*p* < 0.01), 1-on-1 (*p* < 0.01) and 2-on-1 (*p* < 0.01) DM situations (Table 2). Furthermore, the unstandardized beta coefficients indicated that successful tackles were the strongest predictors in TDs (UnB = 1.07), while successful tackles had less influence in 1-on-1 (UnB = 0.72) DM situations, and the least for 2-on-1 DM situations (UnB = 0.33). However, missed tackles were the only predictor for 1-on-1 DM situations (*p* < 0.01). All other match-performance factors did not significantly predict the decisions made (*p* > 0.05).

**Table 2:** Generalized estimating equations with quantity of decision-making as predictors and match-performance factors as dependent variables

Dependent Variables	Predictors	Un-B (95% CI)	SE	*p*	WCS	VIF
TD	Intercept	4.53 (−0.68, 9.73)	2.65	0.09	2.91	–
	Successful Tackles	1.07 (0.75, 1.38)	1.61	<0.01	43.9	1.20
	Missed Tackles	0.73 (−0.08,1.54)	0.41	0.08	3.11	1.20
	Offensive Handling Errors	0.63 (−1.05,2.32)	0.86	0.46	0.54	1.00
1-on-1	Constant	4.10 (0.99,7.22)	1.59	0.01	6.64	–
	Successful Tackles	0.72 (0.44,0.99)	0.14	<0.01	26.7	1.20
	Missed Tackles	0.99 (0.46,1.54)	0.28	<0.01	13.1	1.20
	Offensive Handling Errors	0.05 (−0.69,0.80)	0.38	0.89	0.02	1.00
2-on-1	Constant	0.03	0.90	0.99	0.001	–
	Successful Tackles	0.33 (0.23,0.44)	0.05	<0.01	40.5	1.20
	Missed Tackles	0.03 (−0.31,0.37)	0.17	0.88	0.02	1.20
	Offensive Handling Errors	0.67 (1.48,2.62)	0.41	0.11	2.62	1.00

Un-B: unstandardized beta coefficients with 95% confidence intervals; SE: standard error, WCS: Wald Chi-square, VIF: variance inflation factor

For the generalized linear mixed model of defensive phases (Table 3), the total number of DM situations (i.e., TD) significantly predicted line breaks, committed actions and match-ups (*p* < 0.01) with classification accuracies of 79.7%, 67.3% and 73.2%, respectively. Similarly, 1-on-1 DM situation significantly predicted line breaks, committed action and match-up (*p* < 0.01) with classification accuracy of 76.7%, 62.5% and 73.4%, respectively. However, none of the defensive phases predicted 2-on-1 DM situations (*p* > 0.05), with classification accuracy of 76.7%, 62.5% and 73.4% for line breaks, committed actions and match ups, respectively. When examining whether each position predicted defensive phases within TDs made, wingers significantly predicted line breaks, committed actions and match-ups (*p* < 0.01), whilst centres, halfbacks and backrowers only predicted committed actions (*p* < 0.05). Similar trends were identified within 1-on-1 DM situations, with wingers as significant predictors for line breaks, committed actions and match-ups (*p* < 0.05), although centres were only predictors for line breaks and committed actions (*p* < 0.05). The odds ratio was above one for each defensive phase (i.e., line break, committed action and match-up) during TDs and 1-on-1 DM situations in most positions, suggesting that the odds were greater for these defensive phases to occur in most of these positions. Furthermore, wingers had twice the odds of resulting in a line break than the other positions during TDs and 1-on-1 DM situations, and twice the odds for wingers to result in committed actions than the other positions during 1-on-1 DM situations. However, during 1-on-1 DM situations, halfbacks and backrowers exhibited odds ratios of less than one for line breaks and only backrowers exemplified an odds ratio of less than one for committed actions. In addition, the odds ratios were below one in each defensive phase (i.e., line break, committed action and match-up) for most of the positions, except for backrowers and centres.

## Discussion

The aim of this study was to examine whether position-specific defensive DM situations predicted KPIs in elite rugby league matches. It was hypothesized that the number of decisions made by defensive edge players would impact on match-performance factors, while players closer to the centre of the field (i.e., backrowers and halfbacks) would be exposed to a greater quantity of decisions than positions closer to the sideline (e.g., wingers and centres). The findings of this study demonstrate that successful tackles exhibit a lesser contribution to more complex DM situations (e.g., 2-on-1). Backrowers and halfbacks were found to be exposed to more of these 2-on-1 DM situations compared to wingers and centres. Together these findings highlight the impact of specific DM situations towards successful elite level rugby league gameplay, and that DM demands differ across positions.

### Match-Performance Factors

Successful tackles were significant predictors for all DM situations (TDs, 1-on-1 and 2- on-1), suggesting that a successful tackle is a critical match performance factor during DM situations for edge defenders. However, successful tackles had the least influence on the number of decisions made during 2-on-1 DM situations based on unstandardized beta-coefficients. These findings demonstrate that successful tackles by edge defenders had less bearing on the overall defensive performance outcome as the complexity in defensive match play increased. This 2-on-1 complex DM situation requires edge defenders to anticipate between the different action possibilities of two opposing attackers. For example, as defending players directly perceive the shortening distance between them and their opposing attacker during a 2-on-1 DM situation, a number of different opportunities for the action will emerge and decay (Renshaw et al., 2010). This may include the attacker drawing in the defender in order to pass, or kick, the ball to their unmarked teammate, or create separation between their teammate in order to force the defender to choose which attacking player to defend (i.e., the ball carrier or the oppositng support player). These 2-on-1 DM situations present an array of emergent opportunities for the action, and thus, place greater anticipatory and decision-making demands on the edge-defending player compared to 1-on-1 situations. Based on these findings, coaches are encouraged to create offensive tactical situations to augment 2-on-1 attacking situations, or for defensive tactical situations to minimise 2-on-1 defensive situations during practice.

While successful tackles were important for the overall match performance factor, missed tackles and handling errors exhibited by edge defenders demonstrated limited influence on overall performance outcomes, which were reflective of the entire defensive team. Specifically, when considering that there are five other central playing positions during the defensive play (e.g., hooker, prop, second row forward and lock forward), it is likely that the impact of the quantity and complexity of DM situations had on match performance factors depended on the contribution, and interaction, of all defending players of the team. That is, match-performance factors in the current study were likely the result of a collective effort amongst all defensive players, and their interactions, to generate a shared defensive outcome. Woods et al. (2017) previously reported relative success in predicting the rugby league match outcome based on a unique combination of predominately attacking performance indicators, including try assists, all run meters, offloads, line breaks and dummy half runs. Interestingly, the most notable defensive performance measure related to the game outcome was missed tackles. This may suggest that DM situations during attacking gameplay may be more successful in predicting the game outcome. However, it is important to note that elite rugby league gameplay is highly susceptible to abrupt seasonal changes in predictive KPIs (Woods et al., 2018). Further research investigating the frequency and complexity of DM situations across all defensive positions, and during attacking situations, during different stages of a season is therefore needed.

### Positional Differences

This study also found positional differences when comparing the frequency of 1-on-1 and 2-on-1 DM situations. Backrowers reportedly experienced the greatest number of TD situations, followed by halfbacks and centres who experienced significantly more TD than wingers. This positional trend, from the centre of the field out to the edges, reflects the dynamic nature of a rugby league match, where offensive game-play typically commences from the centre and subsequently transitions to the outer edge of the field (Wheeler et al., 2011). Backrowers are also situated closer to the centre of the field and would therefore likely experience the greatest frequency of 2-on-1 and 1-on-1 DM situations. Successfully preventing the opposing players from scoring or carrying out evasive game play would also minimise the exposure of wingers and centres to 2-on-1 or 1-on-1 situations. Cummins and Orr (2015) previously reported that backrowers exhibited a greater number of tackles than outside back positions (i.e., wingers, centres and halfbacks). Subsequently, the current study supports these findings, whereby backrowers appeared to perform more tackles, likely due to an increased frequency of exposure to DM situations.

The player’s position and the type of DM situation were also found to be predictive of the successfulness of defensive phases that occurred during a play. Exposing more central edge-defensive positions, such as backrowers, did not predict line breaks or unsuccessful match-ups. This may suggest that backrowers are more proficient at anticipating these DM situations, which may be in part due to an increased frequency of DM situations they receive during a game. This conjecture is further supported by backrowers not predicting unsuccessful committed actions during 1-on-1 and 2-on-1 DM situations, possibly because backrowers may exhibit a greater tendency to commit to defensive actions. Interestingly, all other positions significantly predicted unsuccessful committed actions during both total decisions and 1-on-1 DM situations, suggesting that most edge defensive positions avoid making committed defensive actions, and instead, stay relatively fixed in their position, allowing the opposing players to run at them. It has been hypothesised that during complex DM situations, such as those analysed in this study, edge defensive players may be trying to obtain as much visual information as possible before making a final committed decision. This is in line with previous studies suggesting greater delay in committing to an action and reduced anticipatory success during more complex DM situations (Connor et al., 2018a; Gabbett and Abernethy, 2012, 2013).

While backrowers did not predict most defensive phases, wingers and centres significantly predicted line breaks, unsuccessful committed actions and unsuccessful match-ups. These findings together reflect an increase in defensive demands when attacking teams create DM situations for far edge defenders, particularly, wingers and centres. One explanation for this behaviour is to view groups of players as super-organisms who cooperate and coordinate their actions together, often exhibiting swarming behaviours to achieve their common collective goals (Davids et al., 2006; Passos et al., 2013). Players act together in several different interactive subsystems (e.g., offensive players versus defensive players), with self-organisation capabilities and inter-personal coordination tendencies to adapt to evolving environmental constraints during a match (Passos et al., 2009; Vilar et al., 2013). Therefore, the positional demands of wingers suggest they engage with the greatest frequency in these swarming behaviours, drawn in to help their teammates complete a tackle and shut down the opposing attacking players. However, the cost-benefit trade off to this behaviour is increased likelihood of negative defensive phase outcomes such as line breaks if the attacking team creates 1-on-1 or 2-on-1 situations.

The current study has some limitations that warrant further discussion. Playing positions in rugby league are highly consistent, with few exceptions during the game, where players may be caught out of their assigned position. Therefore, the results of this study should be generalised to these specific positions on the field. For example, the winger’s position on the field is always the one closest to the sideline; therefore, the results of the study are generalizable to that position, regardless of the player in that position. Additionally, while TDs constituted a significant predictor of certain defensive phases for specific positions, 2-on-1 and 1-on-1 situations did not reveal a predictive relationship. A possible explanation for this is that the sample size was insufficient for 2-on-1 situations for certain positions. As the purpose of this experiment was to determine whether DM situations warranted further investigation in relation to game performance, this study provides evidence that complex DM situations are in fact an important factor in explaining successful game performances. Future work would benefit from considering the greater exposure to DM situations for backrowers, and the limited number of DM situations experienced by wingers.

## Conclusions

In conclusion, this study revealed that increasing the frequency and complexity of DM situations of edge defenders results in poorer defensive phase performances. Furthermore, there is some evidence that this may also reflect poorer overall team performance and the game outcome. These results emphasize the importance of edge defensive player’s individual roles during complex defensive DM situations. Coaches and practitioners should utilise these findings to improve the representativeness of training to match the demands experienced during the game (Connor et al., 2018b; Pinder et al., 2011). For example, implementing practices using a representative design approach promotes greater fidelity of actions during practice and encourages functionality between perception-action couplings. Strategies for coaches may include implementing small-sided games: (1) with temporary numerical imbalances in the opposing attacker-defender ratio to create 2-on-1 situations and allow players to practice solving the performance problem; and (2) to practice matching-up with opposing players to reduce the occurrences of 2-on-1 situations.
